# An Evaluation Model for Node Influence Based on Heuristic Spatiotemporal Features

**DOI:** 10.3390/e26080676

**Published:** 2024-08-10

**Authors:** Sheng Jin, Yuzhi Xiao, Jiaxin Han, Tao Huang

**Affiliations:** 1 School of Computer Science, Qinghai Normal University, Xining 810016, China; jinsheng.qhnu@gmail.com (S.J.); hanks18165299273@gmail.com (J.H.); taohuang658@gmail.com (T.H.); 2 Qinghai Provincial Key Laboratory of Tibetan Information Processing and Machine Translation, Qinghai Normal University, Xining 810008, China; 3 Key Laboratory of Tibetan Information Processing of Ministry of Education, Qinghai Normal University, Xining 810008, China

**Keywords:** complex networks, node influence, heuristics, higher-order zero model, SIR model

## Abstract

The accurate assessment of node influence is of vital significance for enhancing system stability. Given the structural redundancy problem triggered by the network topology deviation when an empirical network is copied, as well as the dynamic characteristics of the empirical network itself, it is difficult for traditional static assessment methods to effectively capture the dynamic evolution of node influence. Therefore, we propose a heuristic-based spatiotemporal feature node influence assessment model (HEIST). First, the zero-model method is applied to optimize the network-copying process and reduce the noise interference caused by network structure redundancy. Second, the copied network is divided into subnets, and feature modeling is performed to enhance the node influence differentiation. Third, node influence is quantified based on the spatiotemporal depth-perception module, which has a built-in local and global two-layer structure. At the local level, a graph convolutional neural network (GCN) is used to improve the spatial perception of node influence; it fuses the feature changes of the nodes in the subnetwork variation, combining this method with a long- and short-term memory network (LSTM) to enhance its ability to capture the depth evolution of node influence and improve the robustness of the assessment. Finally, a heuristic assessment algorithm is used to jointly optimize the influence strength of the nodes at different stages and quantify the node influence via a nonlinear optimization function. The experiments show that the Kendall coefficients exceed 90% in multiple datasets, proving that the model has good generalization performance in empirical networks.

## 1. Introduction

Complex networks are graphical representations of real-world systems, where nodes and edges represent elements and their interrelationships. In networks, high-influence nodes play a decisive role in the operation and evolution of the system. For example, in social networks, key nodes such as internet celebrities and official accounts can spread information rapidly [1,2]; in power networks, the failure of high-influence nodes may lead to large-scale power outages [3]; and in virus propagation networks, super propagators can accelerate the spread of viruses [4,5]. Therefore, the assessment of node influence has become a focus of recent research; it is considered crucial to reveal the mechanism and functional roles of nodes in networks.

Currently, scholars discussing the issue of node influence primarily utilize the SIR propagation model, focusing on the following aspects: physical topology, node characteristics, and both physical topology and node characteristics.

Regarding approaches based on the physical topology of the network, scholars have developed two types of centrality metric, local and global, to conduct in-depth analyses of the structure and connectivity of networks. Local centrality metrics, such as degree centrality [6], can quickly pinpoint the core nodes in a network, but their view is limited to the direct neighbors of the nodes, and indirect connectivity relationships are ignored. Global centrality metrics, such as the k-shell approach [7], on the other hand, can reveal the network hierarchy, but nodes within the same shell layer are not sufficiently differentiated. To overcome these limitations, researchers have focused on improving the precision and breadth of local metrics [8,9,10] while strengthening the local differentiation abilities of global metrics [11,12,13,14], or proposing new centrality metrics by considering both local and global properties in an integrated manner to more accurately assess the roles and influences of nodes in a network [15,16,17,18]. However, this type of assessment method mainly focuses on the structure and ignores the node characteristics; as a result, the results have low interpretability. Therefore, scholars are turning to influence assessment methods based on node features to improve the accuracy and persuasiveness of the assessment results.

Other methods are based on node features. As the key elements involved in assessing the influence of nodes, node features comprise the inherent attributes and behavioral patterns of nodes. In social networks, these features may include personal attributes such as the users’ age, gender, and education, as well as behavioral patterns such as activity and interaction frequency. Using machine learning techniques, these features can be transformed into quantitative indicators of node influence. The construction of feature engineering is particularly important in this process, which can comprehensively capture the diversity of nodes’ characteristics and, thus, accurately assess their influence [19,20,21,22]. However, methods based on node characteristics often overemphasize the features themselves, neglecting the interactions between nodes, which constrains the accuracy of the assessment. To address this issue, scholars have begun exploring integrated evaluation methods that combine physical topology and node characteristics, aiming to more accurately depict node influence and their interactions

As for node influence evaluation methods based on physical topology and node characteristics, scholars utilize graph neural network (GNN) technology to establish node associations, inputting graph structure information and node features, enabling neural networks to learn network structures and evaluate node influence. However, methods based on graph neural networks (GNNs) [23] often focus excessively on local information and are susceptible to the influence of the network structure. To address this, scholars combine local and global centrality indicators, using models such as graph convolutional networks to aggregate node features and obtain more comprehensive node influence scores [24,25,26,27]. Additionally, due to their advantages in handling sequential data, long short-term memory networks (LSTMs) are widely applied to networks with time series relationships, precisely capturing the dynamic evolution of nodes and demonstrating excellent performance. Inspired by this, scholars have converted graph data into sequence data to fully leverage LSTM models for node influence evaluation [28,29]. However, deep-learning-based methods depend on network carriers, often employing classical network models as training networks [29,30]. These network models may not match empirical network topologies, potentially affecting the model’s evaluation performance across different network structures.

**Figure 1:**
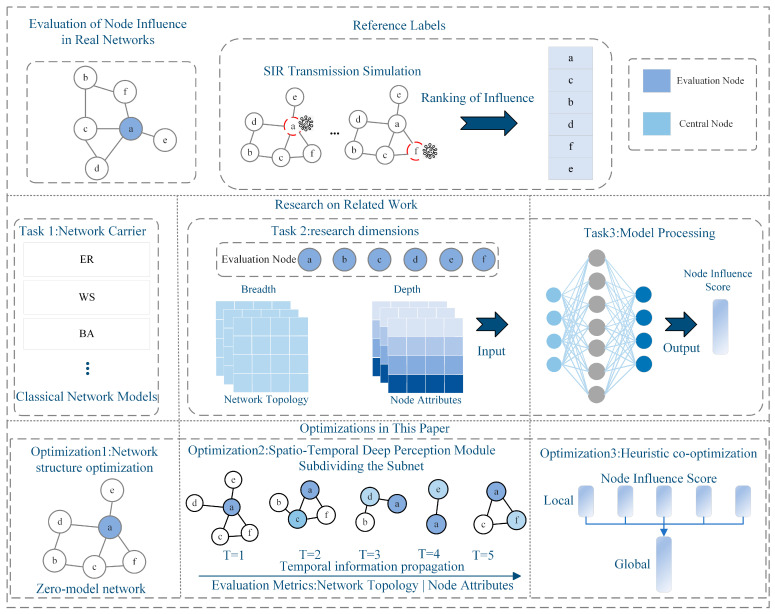
Study overview.

In summary, although these methods have made significant progress in utilizing network structure, node characteristics, or their combined factors to evaluate node influence, they often overlook the dynamic process of node influence from its initial formation to local diffusion and, ultimately, to its impact on the global network. Based on this, this study undertakes the following work, as illustrated in Figure 1.

1. Network structure optimization: We introduce the zero-model method to copy the empirical network topology, reduce the noise interference caused by network structure redundancy, and improve the learning ability of the assessment model for the empirical network topology characteristics.
2. Spatiotemporal depth perception module: This divides the subnetwork and feature construction to enhance the differentiation of node influence, strengthens the spatiotemporal depth of node influence perception, quantifies the node influence, and improves the robustness of the assessment.
3. Heuristic co-optimization: Heuristic evaluation algorithms are used to co-optimize the influence strength of nodes at different stages and quantify the influence of nodes through a nonlinear optimization function.

## 2. Model Description

**Figure 2:**
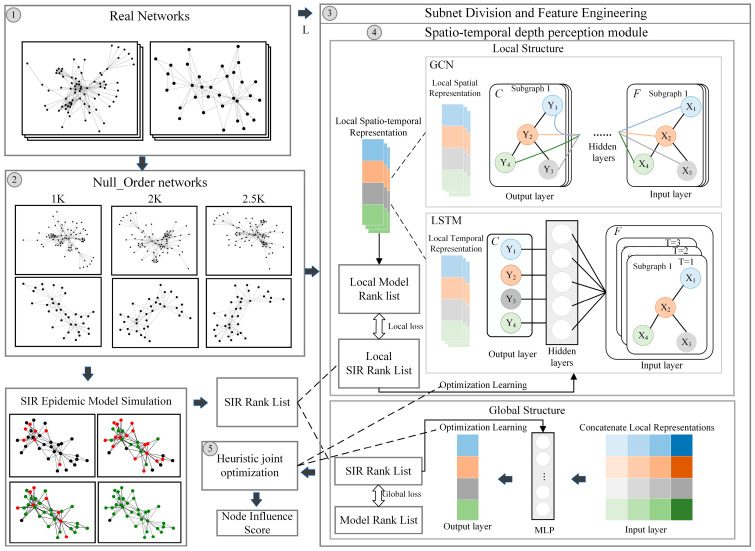
Node influence assessment process diagram.

The HEIST model aims to fuse the spatiotemporal characteristics of nodes for the accurate assessment of nodes’ influence in the process of network dynamic change. The framework is shown in Figure 2, and the specific steps are as follows:

(1) Node influence label construction: Based on the SIR propagation model, the sequence $Rank\in R^{N\times 1}$ of node influence scores is obtained.
(2) High-order zero-model network construction: The zero-model concept is introduced, the empirical network topology is copied to generate the training network G, and the noise perturbation of the network structure redundancy is reduced.
(3) Subnetwork delineation: The network nodes are traversed and the temporal association subnetwork $G_{t_{i}}$ is delineated, centered on each node; $t_{i}$ is the subnetwork evaluation sequence.
(4) Node feature construction: Based on the local subnetwork sequence $G_{t_{1}-t_{N}}$, we use the classical centrality index to characterize the different state neighborhood structures and feature differences of the nodes as the spatial features of the nodes; at the same time, we fill in the results of the subnetwork processed at different moments as the node influence historical a priori information $Y_{v_{i}|G_{t_{1}-t_{N}}}$, which is used as the node influence temporal features.
(5) Spatiotemporal depth perception module: This has a built-in local and global two-layer structure. The local structure uses the GCN network to process node spatial features and the LSTM network to obtain historical information about changes in node influence.
(6) The global structure is based on a heuristic algorithm that analyzes the likelihood of the influence distribution of nodes at different assessment stages and quantifies the node influence on a weighted average basis to achieve joint local and global optimization.

### 2.1. Node Influence Label Construction

The HEIST model uses the SIR propagation model [4] to construct node influence labels, and the SIR model classifies the nodes into three categories, susceptible (*S*), infected (I), and recovered (R), to simulate the process of infectious disease transmission. Initially, one node is infected and the rest are susceptible. In each time interval, the infected person infects the susceptible person in the neighborhood with a certain probability γ and turns to the recovered person directly after the completion of the infection, and the recovered person is no longer infected. The transmission process continues until there are no infected individuals. The number of recovered persons reflects the node’s propagation ability, i.e., its influence C. To simplify the calculation, the recovery rate is set to 1, i.e., infected persons are directly converted to recovered persons. The transmission process is shown in Algorithm 1.
**Algorithm 1:** SIR Propagation**Input:** network G**Output:** $Rank\in {\mathbb{R}}^{N\times 1}$1:   **for** v in G.nodes():2:      R=0, I=1, S=N−1, C=03:      **while** Step>0:4:         **while** I>0:5:          Node I infects neighbors with γ, S → R6:          update I,S7:         **end while**8:         C=C+R, Step=Step−19:      **end while**10:     append C to Rank11:  **end for**

In the algorithm, the input network is G, and output node influence sequence is $Rank\in {\mathbb{R}}^{N\times 1}$. Step 1 is to traverse the network nodes, in turn, as propagation sources. Step 2 is the initialization of the network node state, including the recovery state R, susceptible S, propagator I, and node influence C. Step 3 controls the number of iterations for a single node, and step 4 decides whether to continue the propagation or not, and ends when the infected person in the network is 0. Step 5 is the infection process; the infector I will infect the neighboring nodes with the propagation rate γ, and after infection it becomes the recovery state R. Step 6 updates the node state in the network. Step 7 calculates the node influence by accumulating the number of all restorer states during its iteration. Step 8 adds each node influence to the sequence Rank.

The simulation is performed using the Monte Carlo method. The number of iterations is related to the number of network edges $\left|E\right|$. If $\left|E\right|<100$, it is 100,000 iterations; if $\left|E\right|<10,000$, it is 10,000 iterations; if $\left|E\right|>10,000$, it is 1000 iterations [18]. Finally, the ranked label $Rank\in {\mathbb{R}}^{N\times 1}$ is obtained for the influence of nodes in the network.

### 2.2. Higher-Order Zero-Model Network Construction

The HEIST model treats the network carrier as an undirected unweighted graph containing multiple constraints $G=\{V,E,k,P,P_{j}(k_{1},k_{2}),\bar{C},\bar{C}(k)\}$; $V=\{v_{1},v_{2},\ldots ,v_{N}\}$ is the set of nodes in the graph and $E=\{e_{1},e_{2},\ldots ,e_{N}\}$ is the set of edges in the graph. k is the average degree, P is the degree distribution matrix, $P_{j}(k_{1},k_{2})$ is the joint degree distribution, $k_{1}$ and $k_{2}$ denote the degrees of the two endpoints of a randomly selected edge in the graph, $\bar{C}$ is the average clustering coefficient, and $\bar{C}(k)$ is the average clustering coefficient of the degree correlation.$A={\left[a_{ij}\right]}_{NxN}$ is the neighborhood matrix of the graph, used to describe the network topology, and $a_{\mathrm{ij}}$ is defined as follows:

(1) $$aij=\left\{\begin{array}{c} 1,\;v_{i}\;is\;connected\;to\;v_{j}\\ 0,\;v_{i}\;is\;not\;connected\;to\;v_{j} \end{array}\right..$$

In this study, we introduce the zero-model approach to copy the empirical network and reduce the noise interference due to the redundancy of the network structure; this also improves the learning ability of the evaluation model for the topological properties of the empirical network. The parameters of the constraints in the copying process are shown in Table 1, corresponding to different orders of the zero model.

In the table, the construction of the zero-model network shows a gradual increase in the number of orders with the fitting effect of the empirical network, from the 0th order $G^{0k}$ to retain the number of nodes N and the average degree k, the 1st order $G^{1k}$ to match the node degree distribution P, the 2nd order $G^{2k}$ to expand to the joint degree distribution $P_{j}(k_{1},k_{2})$, the 2.25th order $G^{2.25k}$ to incorporate the average clustering coefficient $\bar{C}$, up to the 2.5th order $G^{2.5k}$, which accurately compounds the system-related average clustering coefficient $\bar{C}(k)$; we use this approach to comprehensively approach the complexity of the empirical network characteristics. Here, we use the 2.5-order zero model as the higher-order zero model, and the 2.5-order zero model is currently the highest-order and practically zero-model network [31,32].

### 2.3. Subnetwork Delineation

The HEIST model evaluates the influence of nodes by dividing the whole network into time-order-associated subnetworks $G_{t_{1}-t_{N}}=\{G_{t_{1}},G_{t_{2}},\ldots ,G_{t_{N}}\}$ with the same number of nodes; $t_{i}$ is the order of evaluation of each subnetwork, which corresponds to the node number in the network, i.e., subnetwork $G_{t_{i}}$ at the moment of $t_{i}$ corresponds to subnetwork $G_{v_{i}}$ with $v_{i}$ as the central node. For a node $v_{i}$, there is a subnetwork $G_{v_{i}}=\{V_{v_{i}}^{\;},E_{v_{i}}^{\;}\}$ centered on it, which contains the set of nodes $V_{v_{i}}^{\;}$ and the set of edges $E_{v_{i}}^{\;}$. The average degree of the top 10% nodes $N_{10\%}$ of the network degree ranking is selected as the local subnetwork size L to fully cover the global network, and the size of the local network L is defined as follows:

(2) $$L=\frac{10}{N}\sum_{i=1}^{N10\%}kvi.$$

For a localized network SubG(vi), its set of nodes node $V_{i}^{local}$ is defined as follows:

(3) $$\left\{\begin{array}{cc} V_{v_{i}}^{\;}=\{v_{i},e_{1}^{v_{i}},e_{2}^{v_{i}},\ldots ,0\}=\{v_{i}\}\cup \{U(v_{i})\cup \underset{L-1-s(v_{i})}{\underbrace{\left\{0,\ldots ,0\right\}}}\} & if\;s(v_{i})<L-1\\ V_{v_{i}}^{\;}=\{v_{i},e_{1}^{v_{i}},e_{2}^{v_{i}},\ldots ,e_{L-1}^{v_{i}}\} & if\;s(v_{i})\geq L-1 \end{array}\right.,$$

(4) $$U(v_{i})=\arg\max u(v_{i})=\{e_{1}^{v_{i}},e_{2}^{v_{i}},\ldots ,e_{n}^{v_{i}}\}.$$

where $u(v_{i})$ is the neighboring node of node $v_{i}$, $U(v_{i})$ is the set of neighboring nodes of node $v_{i}$ in reverse order of their degree value, L is the subnet size, $s(v_{i})$ is the number of neighbors of the node, and $e_{n}^{v_{i}}$ is the neighbor of node $v_{i}$ ranked n in degree.

### 2.4. Node Feature Construction

**Figure 3:**
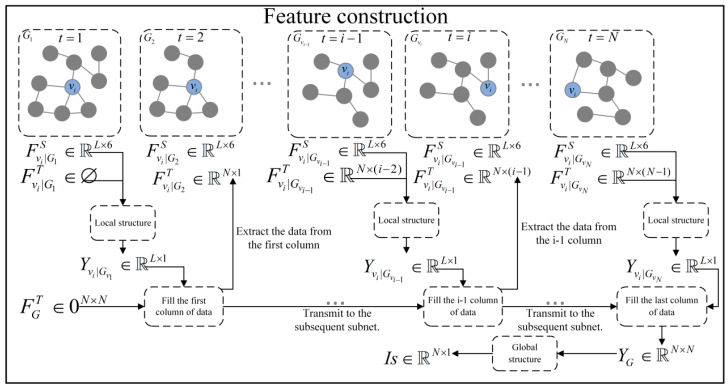
Nodal spatiotemporal feature construction maps.

HEIST is used to investigate node influence based on both temporal and spatial dimensions, with spatiotemporal features constructed around the subnetwork $G_{v_{i}}$, spatial features $F_{v_{i}|G_{v_{i}}}^{S}$ as the node classical centrality indicators, and temporal features $F_{v_{i}|G_{t_{i}}}^{S}$ as the node influence historical a priori information. Details are shown in Figure 3.

Spatial feature construction: This model shows the features of each local subnetwork, and three types of classical node local and global centrality indicator cascades are chosen as the spatial features $F_{v_{i}|G_{v_{i}}}^{S}$ of the nodes in subnetwork $G_{v_{i}}$, as shown in Equation (5). The indicator information is shown in Table 2. For the node-centered local subnetwork $G_{v_{i}}$, the node spatial feature matrix $F_{G_{v_{i}}}^{s}\in {\mathbb{R}}^{L\times 6}$ and the subnetwork topology matrix $Av_{i}$ are used to fully reflect the spatial information related to the subnetwork node influence. For the study network G, the 2D node feature information of each subnetwork is spliced into 3D feature data $F_{G}\in {\mathbb{R}}^{N\times L\times 6}$ for easy model processing.

(5) $$F_{v_{i}|G_{v_{i}}}^{S}=[DC(v_{i|G_{v_{i}}})||EC(v_{i|G_{v_{i}}})||HITS(v_{i|G_{v_{i}}})||CC(v_{i|G_{v_{i}}})||BC(v_{i}{\;}_{|G_{v_{i}}})||Ks(v_{i}{\;}_{|G_{v_{i}}})]$$

Temporal feature construction: Based on the temporal information $t_{1}-t_{i-1}$ of the subgraph sequence $G_{t_{1}-t_{i-1}}$, the temporal features of node $v_{i}$ in subgraph $G_{t_{i}}$ are constructed to further enhance the model’s ability to perceive the temporal dimension by mining the influence of the node’s historical a priori information on the node’s influence. The construction of specific temporal features is shown below:

(6) $$Y_{v_{i}|G_{t_{1}-t_{N}}}=[Y_{v_{i}|G_{t_{1}}}||Y_{v_{i}|G_{t_{2}}}||\cdots ||Y_{v_{i}|G_{t_{N}}}],Y_{v_{i}|G_{t_{i}}}\in Y_{G_{t_{i}}}$$

(7) $$F_{v_{i}|G_{t_{i}}}^{t}=[Y_{v_{i}|G_{t_{1}}}||Y_{v_{i}|G_{t_{2}}}||\cdots ||Y_{v_{i}|G_{t_{i-1}}}],F_{v_{i}|G_{t_{i}}}^{t}\in F_{G_{t_{i}}}^{t}$$

$Y_{G_{t_{i}}}$ is the sequence of feature processing results of subgraphs at different moments as the node influence history prior information $F_{G_{t_{i}}}^{t}$, while $Y_{v_{i}|G_{t_{i}}}$ corresponds to the node’s results in each subgraph and $F_{v_{i}|G_{t_{i}}}^{t}$ corresponds to the node’s timing information in each subnetwork. Here, $Y_{v_{i}|G_{t_{1}-t_{N}}}\in {\mathbb{R}}^{N\times N}$ and $F_{v_{i}|G_{t_{i}}}^{t}\in {\mathbb{R}}^{N\times (i-1)}$.

### 2.5. Spatiotemporal Depth Perception Module

#### 2.5.1. Local Structure

Spatial feature processing involves traversing the network 3D feature data $F_{G}$, extracting one subnetwork node spatial feature $F_{G_{v_{i}}}^{s}$ at a time and combining it with the subnetwork topology information $A_{v_{i}}$ as model inputs, and obtaining the spatial representations of the subnetwork node influence $Y_{G_{v_{i}}}^{s}\in {\mathbb{R}}^{L\times 1}$ through GCN network aggregation. The detailed process is shown in Equation (8):

(8) $$H^{\left(l+1\right)}=\sigma ({\tilde{D}}^{-1/2}\tilde{A}{\tilde{D}}^{-1/2}H^{\left(l\right)}W^{\left(l\right)}).$$

where $H^{\left(l\right)}$ is the node representation matrix of layer l, and $\tilde{A}$ is the augmented adjacency matrix of A+I, where A is the original adjacency matrix and I is the unit matrix, which is used to consider the information of the node itself. $\tilde{D}$ is the diagonal matrix of $\tilde{A}$, where ${\tilde{D}}_{ii}=\sum_{j}{\tilde{A}}_{ij}$, $W^{\left(l\right)}$ represents the weight matrices of layer l, σ is the activation function, and $H^{\left(0\right)}$ is the feature matrix of the input, denoted as $F_{G_{v_{i}}}^{s}\in R^{L\times 6}$. The subnetwork node influence spatial representation $Y_{G_{v_{i}}}^{s}$ serves as the final node representation matrix.

Temporal feature processing involves initializing the two-dimensional zero matrix $F_{G}^{T}=Z^{N\times N}$, which records the historical a priori information related to node influence at each moment; it also processes the spatial features of the subnetwork nodes $F_{G_{v_{i}}}^{s}\in {\mathbb{R}}^{L\times 6}$ via GCN and LSTM processing, using the processed results $Y_{G_{t_{i}}}$ to fill in $F_{G}^{T}$. The node data are filled in on a one-to-one basis, and a column of data is filled in at each moment. The historical a priori information is spliced with the spatial features of the subnetwork at the current moment to form the LSTM input data $F_{G_{t_{i}}}^{T}$. The processing is as follows:

(9) $$i_{t}=\sigma (W_{i}\cdot [h_{t-1},F_{G_{t_{i}}}^{T}]+b_{i}),$$

(10) $$f_{t}=\sigma (W_{f}\cdot [h_{t-1},F_{G_{t_{i}}}^{T}]+b_{f}),$$

(11) $$o_{t}=\sigma (W_{o}\cdot [h_{t-1},F_{G_{t_{i}}}^{T}]+b_{o}),$$

(12) $${\tilde{C}}_{t}=\tanh(W_{c}\cdot [h_{t-1},F_{G_{t_{i}}}^{T}]+b_{c}),$$

(13) $$C_{t}=ft⊙C_{t-1}+it⊙\tilde{C}t;h_{t}=ot⊙\tanh(C_{t}).$$

(14) $$Y_{G}^{T}=\{h1,h2,\ldots hN\};Y_{G_{t_{i}}}^{T}=h_{t}.$$

where σ is the activation function, tanh is the hyperbolic tangent activation function, ⊙ denotes element-by-element multiplication, $F_{G_{t_{i}}}^{T}$ is the input of the current time step, $h_{t-1}$ is the hidden state of the previous time step, W is the integrated weight matrix, and b is the bias vector.$i_{t}$ is an input gate for selectively accepting and storing information about the influence of nodes related to the current time step, and $f_{t}$ is a forgetting gate for selectively forgetting irrelevant historical information. $o_{t}$ is an output gate for deciding which information in the memory cells will be output to the hidden state. ${\tilde{C}}_{t}$ is the candidate memory cell and $h_{t}$ is the hidden state update cell. The output of the LSTM is a sequence of hidden states $h_{t}$. Each subnetwork feature is processed to obtain the corresponding node influence time step representation $Y_{G_{t_{i}}}^{T}$.

Spatiotemporal feature processing: A subnetwork node feature matrix was used to obtain the node influence spatial representation $Y_{G_{v_{i}}}^{s}$ and temporal representation $Y_{G_{t_{i}}}^{T}$ after the batch normalization (*BN*) operation to accelerate the model convergence and to prevent overfitting; finally, the processed spatial and temporal representations are summed up to obtain the spatiotemporal representations of the influence of the nodes of the different subnodes. The details of the processing are shown in Equations (15) and (16):

(15) $$BN(s)=ϒs\frac{s-\mu s}{\sqrt{\sigma_{s}^{2}+\varepsilon }}+\beta s;BN(t)=ϒt\frac{t-\mu t}{\sqrt{\sigma_{t}^{2}+\varepsilon }}+\beta t;t$$

(16) $$Y_{G_{t_{i}}}=BN(Y_{G_{v_{i}}}^{S})+BN(Y_{G_{t_{i}}}^{T})$$

where μs, $\sigma_{s}^{2}$, and μt,$\sigma_{t}^{2}$, respectively, represent the mean and variance for spatial representation s and temporal representation t. Variables s and t correspond to $Y_{G_{v_{i}}}^{s}$ and $Y_{G_{v_{i}}}^{T}$, while ϒs and βs are learnable parameters.

#### 2.5.2. Global Structure

A heuristic evaluation algorithm is used to jointly optimize the strength of the nodes’ influence at different stages and to quantify the nodes’ influence using a nonlinear optimization function. The specific process is shown in Algorithm 2.
**Algorithm 2:** Heuristic Joint Optimization**Input**: $FG\in {\mathbb{R}}^{N\times L\times 6}$, $AG\in {\mathbb{R}}^{N\times L\times L}$, $Rank\in {\mathbb{R}}^{N\times L\times 1}$**Output**: $\mathrm{Is}\in {\mathbb{R}}^{N\times 1}$1:    **while** Epoch > 0:2:       GLoss=0, $F_{G}^{T}\in Z^{N\times N}$3:       **for** $F_{G_{v_{i}}}^{S}$, $A_{G_{v_{i}}}$, $Rank_{G_{v_{i}}}$ in FG, AG, Rank:4:        $F_{G_{v_{i}}}^{S}\in {\mathbb{R}}^{L\times 6}$, $A_{G_{v_{i}}}\in {\mathbb{R}}^{L\times L}$, $Rank_{G_{v_{i}}}\in {\mathbb{R}}^{L\times 1}$, Kdbest=0, LLoss=05:        **while** Lstep>0:6:           $Y_{G_{v_{i}}}^{s}=GCN(F_{G_{v_{i}}}^{S},A_{G_{v_{i}}})$7:           $Y_{G_{t_{i}}}^{T}=LSTM(F_{G}^{T}|F_{G_{v_{i}}}^{S})$,$Y_{G_{v_{i}}}=BN(Y_{G_{v_{i}}}^{s})+BN(Y_{G_{t_{i}}}^{T})$8:           $LLoss=MAE(Y_{G_{v_{i}}},Rank_{G_{v_{i}}})$, $Kd=Kendall(Y_{G_{v_{i}}},Rank_{G_{v_{i}}})$9:           **If** Kd>Kdbest:10:            record $Y_{G_{v_{i}}}$ Record $Y_{G_{v_{i}}}$, fill $F_{G}^{T}$ with $Y_{G_{v_{i}}}$.11:          **end if**12:          Backpropagate the LLoss and update model parameters.13:          Lstep=Lstep−114:       **end while**15:      **end for**16:      $Is=MLP(F_{G}^{T})$17:      GLoss=MAE(Is,Rank)18:      Backpropagate the GLoss and update model parameters.19:      Epoch = Epoch − 120:   **end while**

In the algorithm, the input data $FG\in {\mathbb{R}}^{N\times L\times 6}$, $AG\in {\mathbb{R}}^{N\times L\times L}$, and $Rank\in {\mathbb{R}}^{N\times L\times 1}$ are used to obtain the node influence score $\mathrm{Is}\in {\mathbb{R}}^{N\times 1}$. Step 2 involves global data initialization, containing global loss GLoss and the node influence history information $F_{G}^{T}\in Z^{N\times N}$. Step 3 comprises data extraction, extracting the node spatial features $F_{G_{v_{i}}}^{S}$, subnetwork adjacency matrix $A_{G_{v_{i}}}$, and subnetwork node influence labels $Rank_{G_{v_{i}}}$ for one subnetwork at a time. Step 4 involves local data initialization, containing the most Kendall coefficients Kdbest and the local loss LLoss. Steps 3–11 and 14 are local data initialization. Steps 3–11 and 12–14 are local and global optimization, respectively, forming a heuristic joint optimization. Step 3 is the number of local structural optimizations required to ensure that the large-scale subnetwork can achieve the optimal characterization of node influence within the local area and to enhance the reliability of historical information on node influence. Step 8 records the node influence characterization under the optimal result into the historical information. Step 12 comprises different stages of node influence of the high-dimensional feature data using MLP down to the low-dimensional level, as the node global influence. The specific processing is as follows:

(17) $$Is=W^{\left(K\right)}\sigma (W^{\left(K-1\right)}\sigma (\cdots \sigma (W^{\left(1\right)}x+b^{\left(1\right)})\cdots )+b^{\left(K-1\right)})+b^{\left(K\right)}$$

where $W^{\left(K\right)}$ is the weight matrix of layer K, $b^{\left(K\right)}$ is the bias vector of layer K, σ is the activation function, and x corresponds to YG.

### 2.6. Loss Function

The HEIST model uses the mean absolute error loss (MAE, MAE) as the local and global loss function for joint optimization, which is defined in Equation (18):

(18) $$MAE(y,\hat{y})=\frac{1}{N}\sum_{i=1}^{N}|yi-\hat{y}i|.$$

where $y_{i}$ is the measured value of the ith sample, ${\hat{y}}_{i}$ is the actual value of the ith sample, and $\left|y_{i}-{\hat{y}}_{i}\right|$ is the absolute value of the prediction error of the ith sample.

In the local optimization, y corresponds to the spatiotemporal representation of the influence of subnetwork nodes $Y_{G_{v_{i}}}$, and $\hat{y}$ corresponds to the subseries of subnetwork node influence rankings $Rank_{G_{v_{i}}}$. In the global optimization, y corresponds to the node global influence Is, and $\hat{y}$ corresponds to the network global node influence label Rank.

## 3. Dataset

The dataset used in this study is shown in Table 3; it was sourced from the Network Repository (https://networkrepository.com/index.php (accessed on 15 November 2023)) and the KONECT Project (https://github.com/kunegis/konect-analysis (accessed on 15 November 2023)).

In the table, N represents the network size, E denotes the number of connected edges of the network, K denotes the average degree of the network, L denotes the size of the subnetwork, C denotes the network clustering coefficient, $Ks_{\max}$ denotes the maximum k-kernel degree of the network, and $\beta_{th}$ denotes the network propagation threshold.

## 4. Experimental Analysis

In this section, we first introduce the experimental datasets and evaluation metrics, and this is followed by a series of experiments: (1) correlation analysis, (2) the maximum influence propagation experiment, (3) the visualization experiment using results from small networks, (4) the hyperparameter analysis experiment, and (5) the ablation experiment.

### 4.1. Evaluation Indicators 

In this study, Kendall’s coefficient is used as an indicator to assess the correlation between the predicted and actual rankings, defined as shown in Equation (19):

(19) $$Kendall’tau=\frac{\left(n_{c}-n_{d}\right)}{\frac{1}{2}n(n-1)}$$

where $n_{c}$ is the number of consistent pairs, i.e., pairs whose relative order is consistent for the two variables; $n_{d}$ is the number of inconsistent pairs, i.e., pairs whose relative order is inconsistent for the two variables; and n is the number of samples. Kendall’s coefficient takes values between −1 and 1. Kendall’tau=1 means that the two sequences are in perfect agreement; Kendall’tau=−1 means that the two sequences are in perfect opposition; Kendall’tau=0 means that the two sequences are not correlated.

### 4.2. Baseline Model

The baseline models used in this study are shown in Table 4.

### 4.3. Correlation Analysis

In this section, we compare the HEIST model with other algorithms, setting the infection rate β of the network dataset equal to the propagation threshold $\beta_{th}$. When the infection rate equals the propagation threshold, the system is in a critical state, where each infected node uniformly spreads to other nodes, thereby maintaining relative stability. We calculate the Kendall correlation coefficient between the influence sequences obtained from the HEIST model and the result sequences from the SIR propagation model. Detailed results are shown in Table 5.

Table 5 shows the following:

(1) The performance of the HEIST model is closely related to the scale of subnetwork division; it performs well on datasets such as Karate, Road, Lesmis, Polbooks, Jazz, and PowerGrid, thanks to its dense connectivity relations and high coverage of subnetworks to networks, which enable the model to comprehensively capture the spatial information within the subnetworks and the temporal variations between subnetworks. On the contrary, in scale-free networks such as USAir97 and Email, the degree of front-end nodes is extremely high, which facilitates feature learning, but the large-scale subnetwork division introduces too much filler data and interferes with the model evaluation. However, the model still has advantages over other model-like methods, with the Kendall coefficients all stable above 0.86.
(2) The performance of the HEIST model in Adjnoun networks is affected by low clustering coefficients, which leads to dispersed node relationships and limited tight group formation, which in turn challenges the effectiveness of the local unit feature learning. Nonetheless, the model still outperforms similar methods, highlighting the advantages of modeling based on subnetwork features rather than global features in enhancing the assessment.
(3) When dealing with large networks such as PowerGrid, the HEIST model demonstrates significant advantages over other deep learning methods. The unique distribution of average degree and clustering coefficients in this network challenges the traditional model based on neighboring node feature evaluation, leading to a decrease in the evaluation accuracy. The HEIST model fully captures the network characteristics by setting a smaller subnetwork size and effectively aggregating the local feature information to construct the global features, which results in a better evaluation result.

### 4.4. Analysis of Maximum Influence Node Propagation Experiment

**Figure 4:**
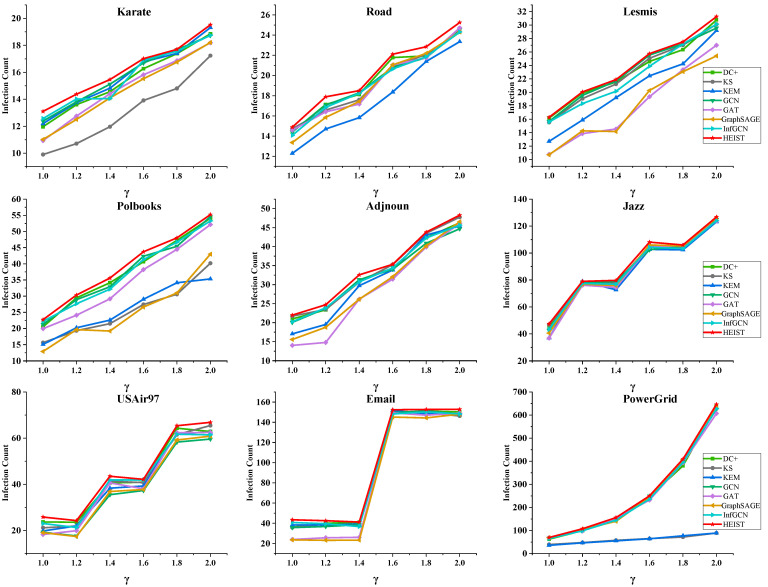
Plot of the scale of impact on the network when the HEIST model is compared to other models with high-impact nodes selected as propagation sources.

To verify the accuracy of the model in obtaining high-influence nodes, we conducted experiments on nine real network datasets of different sizes. The top five highest-influence nodes obtained using eight different methods were defined as high-influence nodes and used as transmission sources for the SIR infectious disease transmission experiments. The infection rates were set as 1, 1.2, 1.4, 1.6, 1.8, and 2.0 times the transmission threshold. The average impact value of the high-influence nodes selected using each method on the network size was calculated via a Monte Carlo simulation of a large number of transmission experiments. To simplify the experiments, the recovery rate of the SIR model was set to 1, i.e., a node becomes immune immediately after propagation. The number of immune nodes at the end of propagation is considered to be an indicator of the influence size. The specific results are shown in Figure 4.

The figure shows that the high-influence nodes identified by the HEIST model have the most significant impact on nine real networks of different sizes when acting as propagation sources. This means that the top five high-influence nodes evaluated by the model are closest to the super-propagators in the propagation mechanism. This phenomenon indicates that the model is efficient and discriminative in identifying front-end nodes; such nodes are contained in multiple spatially information-rich subnetworks with excellent feature learning.

### 4.5. Visualization of Experiments on Small Networks

**Figure 5:**
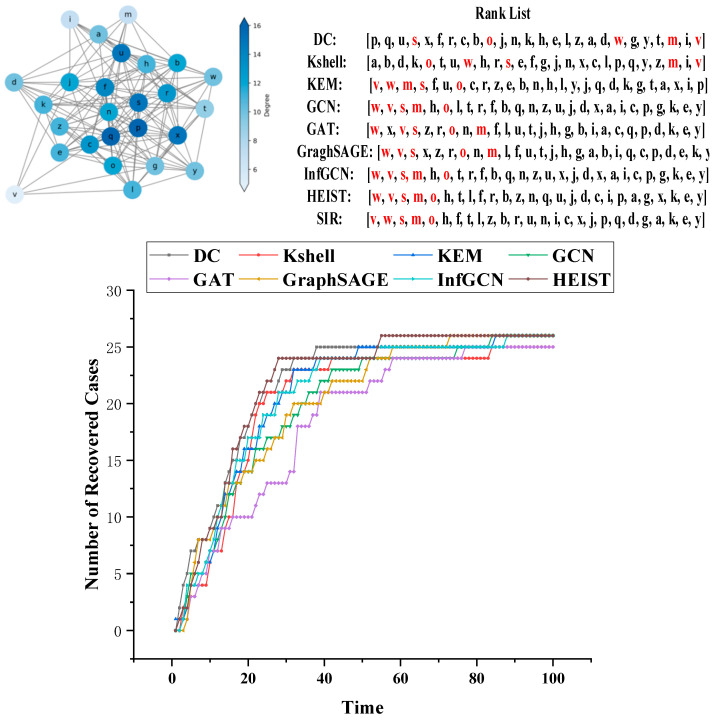
Analysis of propagation in a small network.

To gain a clearer understanding of the model’s evaluation of the top five nodes in terms of influence, a small network with 26 nodes was generated for visualization and analysis. Eight different methods were used to evaluate the node influence in the network. The top five nodes with the highest influence were selected as sources for the SIR propagation model to observe the model’s effectiveness, as shown in Figure 5.

Figure 5 consists of three parts: the network topology visualization intuitively displays the degree of nodes, differentiated by color; the influence assessment comparison covers eight types of methods and SIR simulations, with the top five high-influence nodes marked in red; and the propagation simulation experiments are based on the top five nodes selected using each method, in order to visualize the propagation effect from the two dimensions of speed and scale. The results show that the model in this study exhibits outstanding performance in identifying the key front-end nodes, which can be used as the source to quickly infect the whole network, due to the rich features and pure neighborhood information of these nodes, which gives full play to the advantages of the model.

### 4.6. Hyperparametric Analysis

#### Analysis of Local Structural Parameters

HEIST introduces the heuristic idea of advancing global continuity from local ordering, so it is crucial to ensure the accuracy of the local node influence assessment. In this study, different local structure execution times are selected for the experiments, and the infection rate is selected as the propagation threshold $\beta_{th}$. The score sequence obtained from the model is compared with the infection sequence of the SIR propagation model, and the Kendall coefficient is calculated. Detailed results are shown in Table 6.

As shown in Table 6, the number of executions of the local structure significantly affects the final evaluation results, where the optimal effectiveness is closely related to the number of executions and the local cell size. For datasets with a large local size L, such as Jazz, USAir97, Email, etc., the model needs to increase the number of local executions to optimize its effectiveness; on the contrary, in networks with many nodes but a small local size L setting, such as PowerGrid, the model only needs a single local execution to achieve the optimal performance, which reflects the model’s adaptability and robustness under different network structures.

### 4.7. Ablation Experiment

#### 4.7.1. Training Network Ablation Experiment

**Figure 6:**
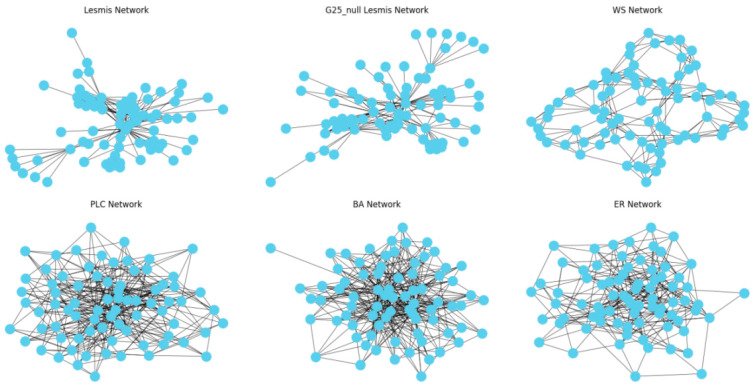
Visualization of different network structures.

This study uses six types of datasets, including ER random networks, BA scale-free networks, WS small-world networks, PLC power-law cluster networks, and various order null model networks. These datasets have the same node size, average degree, and local structure as the original network. They were used as training sets for the model, while real network datasets were used for testing. As an example, the network structure of the Les Misérables real dataset was visualized, as shown in Figure 6.

**Figure 7:**
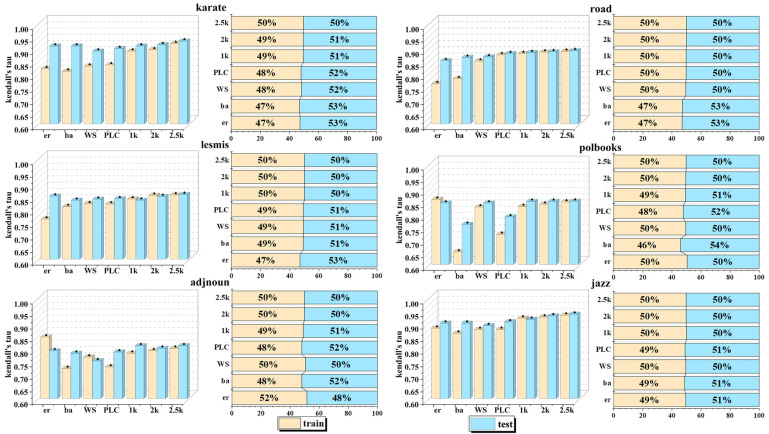
Graph of the effect of different training network training tests.

As shown in the figure, compared to other network models, null model networks closely match the structure of the original network. To demonstrate the impact of training and testing network structures on evaluation results, different training networks are tested, with the results shown in Figure 7.

The bar chart in the figure compares the performance of different training network models in the training and testing phases and visualizes the difference in the training–testing ratio through the right-hand illustration. The analysis shows the following:

(1) The training results of the training networks based on ER stochastic network, BA scale-free network, WS small-world network, and PLC power-law cluster network are significantly affected by the randomness of the generation rules and show a degree of uncertainty. On the contrary, as the order of the zero-model network increases, the constraints increase, the training network gradually approaches the real network characteristics, and the training test results tend to be stable, indicating that the model learns more accurate features.
(2) ER networks have limited internode variability, which limits the model’s ability to capture the subtle features of complex networks, leading to significant differences between the training and testing results. WS networks, on the other hand, exhibit clear association structures with significant internode variability, but the phenomenon of overlapping associations may increase the training complexity and be affected by the structure of the real network, e.g., in the adjnoun network (with a low clustering coefficient of 0.17). Using high-clustering WS network training may lead to poor test performance.
(3) In power-law distribution networks such as BA scale-free networks and PLC power-law cluster networks, the scarcity of highly influential nodes means that the selected scale L often exceeds the average degree range when dividing the local network, resulting in a large amount of filler data, introducing noise to the model, and affecting the stability of the test results. In particular, local networks centered on end nodes may affect the overall training effect due to the loss of feature information, a phenomenon reflected in several datasets such as karate, road, polbooks, and adjnoun.

#### 4.7.2. Spatiotemporal Feature Ablation Experiment

The HEIST model fuses the GCN and LSTM models to obtain the spatial representation of network node influence using the GCN model and the temporal correlation of node influence in different subnets using the LSTM model. In order to verify the feasibility of the model, ablation experiments are carried out, where separate GCN and LSTM models are used for local-to-global learning to explore the influence of hybrid spatiotemporal features on the model effect. The specific experimental results are detailed in Table 7.

In this experiment, the HEIST model uses data that are locally executed once; it shows significant advantages in assessing node influence by fusing the GCN and LSTM models. The HEIST model significantly improves the assessment accuracy in all the tests on nine different datasets. The analysis shows that, when relying only on spatial features, it is possible to identify local node influence, but this method lacks global vision; meanwhile, focusing only on temporal features makes it difficult to ensure local accuracy, which in turn introduces larger errors into the global fusion. The success of the HEIST model lies in its heuristic learning mechanism, which emphasizes local ordering to drive global continuity and ensures the comprehensiveness and accuracy of the assessment. Local instability or global discontinuity can weaken the model, highlighting the importance of integrating the spatial and temporal features of node influence.

## 5. Conclusions

Node influence recognition has practical value in the fields of social networks, communication, and disease transmission; it helps to accurately identify key nodes to optimize information dissemination strategies and disease prevention and control measures. In this study, we introduce a high-order zero model as a training network, deconstruct the static network into multiple time-ordered correlated subnetworks, and comprehensively consider the spatiotemporal characteristics of each subnetwork; a heuristic joint optimization algorithm is applied to quantify the global influence of nodes through local ordering to drive global continuity. The experiments prove that the method performs well. This effectiveness is attributed to the strategy of exchanging space for time, but this also leads to high levels of time complexity. Future research will be devoted to exploring new feature construction methods, more reasonable subnet divisions, and more efficient models to deepen the study of node influence.

## Figures and Tables

**Table 1:** Correlation table of constraints for different orders of zero models.

Zero Model	Restrictive Condition
$G^{0k}$	N, k.
$G^{1k}$	P
$G^{2k}$	$P_{j}(k_{1},k_{2})$
$G^{2.25k}$	$\bar{C}$
$G^{2.5k}$	$\bar{C}(k)$

**Table 2:** Descriptions of the selected features.

Features	Descriptions
Degree centrality (*DC*)	Measures the number of direct connections a node has in the network and reflects the direct influence and activity of the node.
Eigenvector centrality (*EC*)	Measures the pattern of connections between a node and its neighboring nodes and takes the importance of the neighboring nodes into account.
*HITS*	Measures the importance of the node as a source of information and information disseminator in the network.
Closeness centrality (*CC*)	Measures the total length of the shortest path between a node and other nodes and describes the role of a node as a broadcaster in the network.
Betweenness centrality (*BC*)	Measures the number of shortest paths through a node and describes the node’s role as a bridge in the network.
K-shell (*Ks*)	Measures the structural position of a node in the network and describes the node’s central role in the network.

**Table 3:** Network statistical characteristics.

Network	N	E	K	L	C	$Ks_{\max}$	$\beta_{th}$
Karate	34	156	9.18	15	0.57	8	0.13
Road	39	170	8.72	26	0.45	6	0.08
Lesmis	77	254	6.6	20	0.57	9	0.08
Polbooks	105	441	8.4	21	0.49	6	0.08
Adjnoun	112	850	15.18	22	0.17	12	0.07
Jazz	198	2742	27	62	0.62	30	0.03
USAir97	332	2126	12.8	64	0.63	27	0.02
Email	908	10430	22	117	0.49	84	0.01
PowerGrid	4941	6594	2.67	6	0.08	5	0.26

**Table 4:** Baseline model characteristics.

Baseline Model	Method Description
K-shell [7]	Quantifies the influence of a node by assigning it to different shell levels. No des within the same shell have the same core value, i.e., they have similar position and importance in the network. The higher the shell value of a node, the more significant its core position in the network structure and the higher its influence.
DC+ [8]	Comprehensively assesses the node influence by combining the degree of the node itself and the degree of its neighboring nodes.
KEM [14]	Calculates the K-order information entropy of nodes in the network as the node influence score.
InfGCN [25]	Learns representations of nodes by combining neighboring graphs and classical structural features as input. These representations contain the structural and feature information of the nodes, which can reflect the importance and influence of the nodes in the process of virus propagation or information dissemination.
GCN [26]	Learns the embedding representation of a node by aggregating its neighbor information. These embedding vectors fuse the local and global information of a node, which can reflect the node’s position, role, and relationship with other nodes in the network.
GraphSAGE [27]	An inductive learning approach is used to generate the embedding representation of a node by sampling and aggregating the information of its neighboring nodes. This approach both considers the local information of the nodes and captures the global structure of the network.

**Table 5:** Comparison of Kendall’s correlation coefficients between the HEIST model and other models.

Dataset	Network Characteristic				Methods						
	*N*	K	L	C	DC+	KS	KEM	GCN	Graph SAGE	Inf GCN	HEIST
Karate	34	9.18	15	0.57	0.79	0.80	0.783	0.803	0.817	0.80	**0.946**
Road	39	8.72	26	0.45	0.89	0.677	0.908	0.843	0.873	0.839	**0.924**
Lesmis	77	6.6	20	0.57	0.864	0.801	0.864	0.878	0.864	0.868	**0.8** **8**
Polbooks	105	8.4	21	0.49	0.841	0.746	0.859	0.801	0.854	0.798	**0.873**
Adjnoun	112	15.2	22	**0.17**	0.872	0.83	**0.8** **81**	0.82	0.847	0.854	0.853
Jazz	198	27	62	0.62	0.947	0.81	0.954	0.74	0.915	0.925	**0.95** **8**
USAir97	332	**12.8**	**64**	0.63	0.899	0.80	**0.** **913**	0.86	0.861	0.899	0.886
Email	908	22	117	0.49	0.79	0.736	**0.** **933**	0.764	0.637	0.788	0.799
PowerGrid	4941	**2.** **7**	**6**	**0.08**	0.709	0.606	0.731	0.43	0.44	0.714	**0.75** **9**

**Table 6:** Effect of the number of executions of the local model.

Network	L	β	HEIST1	HEIST3	HEIST5	HEIST7	HEIST10	HEIST12
Karate	**15**	0.13	0.917	**0.946**	0.934	0.938	0.935	0.938
Road	**26**	0.08	0.891	0.896	0.92	**0.9** **24**	0.922	0.911
Lesmis	**20**	0.08	0.858	0.861	**0.8** **81**	0.867	0.866	0.865
Polbooks	**21**	0.08	0.852	0.855	**0.873**	0.863	0.865	0.861
Adjnoun	**22**	0.07	0.819	0.823	0.824	**0.85** **3**	0.843	0.85
Jazz	**62**	0.03	0.936	0.936	0.938	0.948	**0.95** **8**	0.947
USAir97	**64**	0.02	0.802	0.817	0.833	0.856	**0.8** **86**	0.869
Email	**117**	0.01	0.701	0.713	0.737	0.746	0.765	**0.79** **9**
PowerGrid	**6**	0.26	**0.7** **59**	0.747	0.742	0.745	0.748	0.745

**Table 7:** Ablation experiment effect graph.

Network	L	β	GCN	LSTM	HEIST
Karate	15	0.13	0.865	0.9108	**0.917**
Road	26	0.08	0.8678	0.8866	**0.8** **914**
Lesmis	20	0.08	0.8421	0.8454	**0.8578**
Polbooks	21	0.08	0.8359	0.76	**0.8520**
Adjnoun	22	0.07	0.8072	0.8108	**0.8191**
Jazz	62	0.03	0.928	0.8923	**0.9357**
USAir97	64	0.02	0.7681	0.7408	**0.8015**
Email	117	0.01	0.6652	0.6725	**0. 7012**
PowerGrid	6	0.26	0.7363	0.7215	**0.7585**

## Data Availability

The data that support the findings of this study are available upon request from the corresponding author. The data are not publicly available due to ongoing follow-up research.

## References

[1] Belfin R.V., Bródka P. (2018). Overlapping community detection using superior seed set selection in social networks. Comput. Electr. Eng..

[2] Chandran J., Viswanatham V.M. (2022). Dynamic node influence tracking based influence maximization on dynamic social networks. Microprocess. Microsyst..

[3] Huang J., Xiong M., Wang J. (2022). Route choice and parallel routes in subway Networks: A comparative analysis of Beijing and Shanghai. Tunn. Undergr. Space Technol..

[4] Kermack W.O., McKendrick A.G. (1991). Contributions to the mathematical theory of epidemics--I. 1927. Bull. Math. Biol..

[5] Fu L., Yang Q., Liu Z., Liu X., Wang Z. (2022). Risk identification of major infectious disease epidemics based on complex network theory. Int. J. Disaster Risk Reduct..

[6] Gao S., Ma J., Chen Z., Wang G., Xing C. (2014). Ranking the spreading ability of nodes in complex networks based on local structure. Phys. A Stat. Mech. Its Appl..

[7] Kitsak M., Gallos L.K., Havlin S., Liljeros F., Muchnik L., Stanley H.E., Makse H.A. (2010). Identification of influential spreaders in complex networks. Nat. Phys..

[8] Chen D., Su H. (2023). Identification of influential nodes in complex networks with degree and average neighbor degree. IEEE J. Emerg. Sel. Top. Circuits Syst..

[9] Flores J., Romance M. (2018). On eigenvector-like centralities for temporal networks: Discrete vs. continuous time scales. J. Comput. Appl. Math..

[10] Zhang H., Zhong S., Deng Y., Cheong K.H. (2021). LFIC: Identifying influential nodes in complex networks by local fuzzy information centrality. IEEE Trans. Fuzzy Syst..

[11] Yuan H.L., Feng C. (2022). Ranking and Recognition of Influential Nodes Based on k-shell Entropy. Comput. Sci..

[12] Yang X., Xiao F. (2021). An improved gravity model to identify in-fluential nodes in complex networks based on K-shell method. Knowl. Based Syst..

[13] Chawla P., Mangal R., Chandrashekar C.M. (2020). Discrete-time quantum walk algorithm for ranking nodes on a network. Quantum Inf. Process..

[14] Zhao X., Yu H., Huang R., Liu S., Hu N., Cao X. (2023). A novel higher-order neural network framework based on motifs attention for identifying critical nodes. Phys. A Stat. Mech. Its Appl..

[15] Yang S., Zhu W., Zhang K., Diao Y., Bai Y. (2024). Influence Maximization in Temporal Social Networks with the Mixed K-Shell Method. Electronics.

[16] Xi Y., Cui X. (2023). Identifying Influential Nodes in Complex Networks Based on Information Entropy and Relationship Strength. Entropy.

[17] Yu Y., Zhou B., Chen L., Gao T., Liu J. (2022). Identifying Important Nodes in Complex Networks Based on Node Propagation Entropy. Entropy.

[18] Wu Y., Ren Y., Dong A., Zhou A., Wu X., Zheng S. (2024). Key Nodes Identification Method Based on Neighborhood K-shell Distribution. Comput. Eng. Appl..

[19] Inuwa-Dutse I., Liptrott M., Korkontzelos I. (2018). Detection of spam-posting accounts on Twitter. Neurocomputing.

[20] Zhao G., Jia P., Huang C., Zhou A., Fang Y. (2020). A machine learning based framework for identifying influential nodes in complex networks. IEEE Access.

[21] Wen X., Tu C., Wu M., Jiang X. (2018). Fast ranking nodes importance in complex networks based on LS-SVM method. Phys. A Stat. Mech. Its Appl..

[22] Qiu L., Zhang J., Tian X. (2021). Ranking influential nodes in complex networks based on local and global structures. Appl. Intell..

[23] Zhang M., Wang X., Jin L., Song M., Li Z. (2022). A new approach for evaluating node importance in complex networks via deep learning methods. Neurocomputing.

[24] Fan C., Zeng L., Ding Y., Chen M., Sun Y., Liu Z. Learning to identify high betweenness centrality nodes from scratch: A novel graph neural network approach. Proceedings of the 28th ACM International Conference on Information and Knowledge Management.

[25] Zhao G., Jia P., Zhou A., Zhang B. (2020). InfGCN: Identifying influential nodes in complex networks with graph convolutional networks. Neurocomputing.

[26] Qu H., Song Y.-R., Li R., Li M. (2023). GNR: A universal and efficient node ranking model for various tasks based on graph neural networks. Phys. A Stat. Mech. Its Appl..

[27] Hamilton W.L., Ying R., Leskovec J. Inductive Representation Learning on Large Graphs. Proceedings of the 31st International Conference on Neural Information Processing Systems (NIPS’17).

[28] Kumar S., Mallik A., Panda B.S. (2023). Influence maximization in social networks using transfer learning via graph-based LSTM. Expert Syst. Appl..

[29] Zhu J., Wang L. (2021). Identifying influential nodes in complex networks based on node itself and neighbor layer information. Symmetry.

[30] Xi Y., Wu X., Cui X. (2024). Node Influence Ranking Model Based on Transformer. Comput. Sci..

[31] Gjoka M., Kurant M., Markopoulou A. 2.5 k-graphs: From Sampling to Generation. Proceedings of the 32nd IEEE International Conference on Computer Communications.

[32] Mahadevan P., Hubble C., Krioukov D., Huffaker B., Vahdat A. Orbis: Rescaling degree correlations to generate annotated internet topologies. Proceedings of the 2007 Conference on Applications, Technologies, Architectures, and Protocols for Computer Communications.

